# All-positive HBsAg, HBsAb, HBeAg, HBeAb, and HBcAb in a patient with hepatitis B: A case report

**DOI:** 10.1097/MD.0000000000041197

**Published:** 2025-01-10

**Authors:** Fenfang Wang, Qianchun Zhang, Ju Zhang, Penghui Yang, Fei Pan, Xuefang Ren, Hang Yuan, Zhongbao Chang

**Affiliations:** a Department of Clinical Immunology, Nanjing Kingmed Clinical Laboratory, Nanjing, Jiangsu, China; b Suqian Maternity Hospital, Suqian, Jiangsu, China.

**Keywords:** hepatitis B, liver function, positive, serum markers

## Abstract

**Rationale:**

Mass vaccination, low cost of immunoglobulins, and new drugs led to the emergence of new, unusual patterns of hepatitis B serum markers. This study reported a rare case of hepatitis B with all 5 positive serum markers, including HBsAg, HBsAb, HBeAg, HBeAb, and HBcAb.

**Patient concerns:**

A 30-year-old female patient was admitted due to abnormal liver function. The 5 serum markers were all positive (+), including HBsAg, HBsAb, HBeAg, HBeAb, and HBcAb. After antiviral therapy, she was discharged with normal liver function and decreased hepatitis B virus-DNA levels, but all 5 serum markers were still positive. Regular follow-up was conducted every 3 months.

**Diagnoses:**

Abnormal liver function.

**Interventions:**

The patient received antiviral treatment and liver protection therapy using entecavir dispersive tablet 0.5 mg po qd and glycyrrhizate diamine enteric capsule 150 mg po tid.

**Outcomes:**

The patient’s liver function was normal, hepatitis B virus-DNA continued to decline, and HBeAb turned negative at 6 months. After 9 and 12 months of follow-up, the results of hepatitis B markers in the patient were stable in HBsAg positive (+), HBeAb positive (+), and HBcAb positive (+).

**Lessons:**

The rare pattern of all 5 positive hepatitis B markers may occur in patients with chronic hepatitis B. Improvements can be achieved using first-line drugs and conventional treatment. Nevertheless, more attention should be paid to the patient’s condition.

## 1. Introduction

There are 32 patterns for the 5 serum markers of hepatitis B commonly used in practice, each pattern having a different clinical significance.^[1]^ Due to vaccination, the low price of immunoglobulins, and the marketing of new drugs, many patients have occult hepatitis B virus (HBV) infection,^[2]^ and rare hepatitis B serological patterns are beginning to appear in clinical practice. For instance, the “5-positive” pattern of hepatitis B is rare, and that particular pattern appears to be different from the other patterns in terms of diagnosis, treatment, and prognosis.^[1]^ The laboratory is often at a loss when reporting rare patterns of hepatitis B due to the lack of relevant experience. Even if the results are confirmed, and the report is issued, the clinicians will also be confused about the examination results and may even doubt their accuracy. Therefore, laboratory personnel and clinicians should be aware of the possibility of such results. This report presents a rare case of hepatitis B with all 5 serum markers positive, including HBsAg, HBsAb, HBeAg, HBeAb, and HBcAb.

## 2. Case report

A female patient, 30 years old, with chronic hepatitis B for 15 years, was admitted to the Department of Infectious Diseases of the Suqian Maternity Hospital, on May 24, 2021, due to abnormal liver function markers (elevated alanine aminotransferase [ALT] and aspartate aminotransferase [AST]) found in physical examination 2 days earlier. The patient had received interferon antiviral therapy 6 months earlier for chronic hepatitis B. Repeated tests showed abnormal liver function and suggested possible cirrhosis. Laboratory examination showed ALT at 431 U/L (7–40 U/L), AST at 262 U/L (13–35 U/L), and γ-glutaryl transferase (GGT) at 135 U/L (7–45 U/L). Surprisingly, the 5 serum markers of hepatitis B were all positive (+), including HBsAg, HBsAb, HBeAg, HBeAb, and HBcAb (Table 1).

**Table 1:** Detection of liver function, hepatitis B 5 serum markers and HBV-DNA.

Serum markers	First detection and reexamination		Second detection and reexamination		Discharge examination	3-month follow-up	6-month follow-up	9-month follow-up	12-month follow-up
	CLIA	Ultracentrifugation	CLIA	ELISA	CLIA	CLIA	CLIA	CLIA	CLIA
HBsAg	12,125.67 (+)	12,381.58 (+)	12,601.35 (+)	(+)	12,033.60 (+)	12,165.38 (+)	12,511.78 (+)	12,403.06 (+)	12,621.45 (+)
HBsAb	31.20 (+)	30.71 (+)	30.20 (+)	(+)	26.71 (+)	25.76 (+)	20.71 (+)	9.71 (−)	5.71 (−)
HBeAg	1.82 (+)	1.77 (+)	1.75 (+)	(+)	1.88 (+)	1.65 (+)	1.57 (+)	0.87 (−)	0.77 (−)
HBeAb	0.87 (+)	0.90 (+)	0.82 (+)	(+)	0.89 (+)	0.92 (+)	1.86 (−)	0.83 (+)	0.76 (+)
HBcAb	9.78 (+)	10.36 (+)	10.28 (+)	(+)	10.73 (+)	10.55 (+)	10.36 (+)	9.76 (+)	10.25 (+)
HBV-DNA (IU/mL)	5.45 × 10^4^		5.20 × 10^4^		1.55 × 10^4^	8.80 × 10^3^	4.20 × 10^3^	2.25 × 10^3^	8.20 × 10^2^
ALT (U/L)	431		402		38	35	30	32	28
AST (U/L)	262		253		32	30	28	30	25
GGT (U/L)	135		132		30	33	25	36	33

ALT = alanine aminotransferase, AST = aspartate aminotransferase, CLIA = chemiluminescence immunoassay, ELISA = enzyme-linked immunosorbent assay, GGT = γ-glutamyl transferase, HBV-DNA = hepatitis B virus deoxyribonucleic acid.

**Figure 1.**
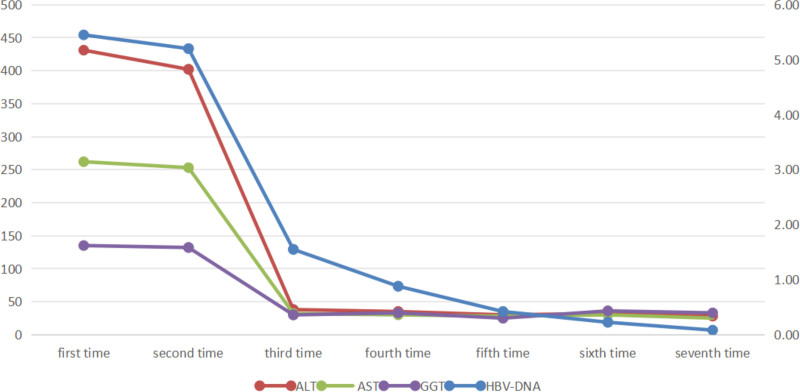
Dynamic changes of the 5 serum markers for hepatitis B. ALT = alanine aminotransferase, AST = aspartate aminotransferase, GGT = γ-glutamyl transferase, HBV-DNA = hepatitis B virus deoxyribonucleic acid.

Then, the patient received antiviral treatment and liver protection therapy using entecavir dispersive tablet 0.5 mg po qd and glycyrrhizate diamine enteric capsule 150 mg po tid. She was discharged with normal liver function and decreased hepatitis B virus deoxyribonucleic acid (HBV-DNA) levels, while all 5 serum hepatitis B markers were still positive. The 3- and 6-month follow-up results of hepatitis B virus serum markers showed that HBeAb turned negative at 6 months, while the other 4 markers remained positive.However,the 9-month follow-up results of hepatitis B virus serum markers showed that both anti-HBs and HbeAg turned negative while anti-HBe turned positive, and this common outcome pattern of HBsAg (positive [+]), anti-HBs (negative [−]), HBsAg (negative [−]), anti-HBe (positive [+]), and anti-HBc (positive [+]) began to emerge and remained stable until the 12-month follow-up (the last test; Table 1). The dynamic changes of the 5 serum markers of hepatitis B after hospitalization and treatment are shown in Figure 1. During the 6-month follow-up after discharge, the patient’s condition was stable and continued to improve. Entecavir performed well as a first-line antiviral therapy.

## 3. Discussion

Thirty-two patterns of the 5 serum markers of hepatitis B commonly used in practice can theoretically be observed, each pattern having a different clinical significance and treatment approach.^[1]^ Simultaneously positive HBsAg and HBsAb is an atypical pattern that could be related to the long-term use of hepatitis drugs and associated with a higher frequency of genetic variations in the pre-S/S region.^[3]^ Antibodies against HBV components indicate active immunity against HBV infection.^[4]^ Still, the presence of high levels of HBV antigens indicates active virus replication but also inefficient neutralization by the antibodies, probably due to immune escape mutations.^[5]^ Immune pressure in patients with HBV infection can lead to mutants being able to escape the immune system.^[5]^

Qualitatively, being positive for HBsAg indicates the presence of HBV. Being positive for HBsAb indicates successful vaccination with the HBV vaccine or the existence of HBV antibodies. HBeAg-positive indicates that HBV replicates actively, and the blood is highly infectious. Being positive for HBeAb indicates that the replication of HBV has changed from an active state to a relatively static state. HBcAb-positive indicates that the patient has been or is infected with HBV.^[1,6]^ Heavy drinking will promote the loss of liver immune functions and allow HBV to persist chronically.^[7,8]^ Immune cells and viruses are no longer in balance under acute stress, resulting in a situation in which all 5 serum markers are positive. In the case reported here, the results did not change much after 6 months of therapy, suggesting that the drug selected a mutant HBV with a resistance gene or the derivation of new variants. Still, the HBV-DNA levels decreased significantly after half a year of treatment. Therefore, the authors believe that HBV-DNA has more monitoring significance when clinicians want to evaluate a treatment regimen’s effects. In addition, many reports have studied the relationship between HBsAg and HBsAb double positivity and Pre-S/S region gene mutations in chronic hepatitis B patients,^[9,10]^ but further analysis was not performed in the case reported here because the laboratory did not carry out relevant gene mutation detection tests.

As the testing equipment technology is constantly being improved and now allows the fast detection of all 5 hepatitis B serum markers, it is possible that more patients with paradoxical patterns will be identified soon, the study by Hashim et al^[11]^ found that 2.8% (2/70) of patients were positive for the coexistence of HBeAg and HBeAb, the retrospective study by Xu et al^[12]^ showed a considerable prevalence of coexistence of HBsAg and anti-HBs in chronic HBV-infected children, and the management of such patients is unknown. In the case reported here, treatment with antiviral therapy was successful in decreasing the HBV-DNA levels for now, but the exact strategy to be used in the future is unknown. Nevertheless, because of the risk of false positive results, repeat testing should be performed using different test methods. In case of clinically rare abnormal results, it is necessary to communicate with the clinician in time to solicit the patient for resampling to eliminate the possibility of manipulation errors or specimen contamination. It is also suggested to document adequately all tests and retests performed and to test for HBV-DNA. Ideally, mutations should also be examined.

In conclusion, laboratories encountering surprising hepatitis B abnormal test results should confirm the results using other methods, when possible, and timely communicate with the clinician.

## Author contributions

**Conceptualization:** Fenfang Wang, Qianchun Zhang.

**Data curation:** Fenfang Wang, Qianchun Zhang.

**Formal analysis:** Ju Zhang, Penghui Yang, Fei Pan, Xuefang Ren, Hang Yuan, Zhongbao Chang.

**Funding acquisition:** Penghui Yang, Fei Pan, Xuefang Ren, Zhongbao Chang.

**Investigation:** Fenfang Wang, Ju Zhang, Penghui Yang, Fei Pan, Xuefang Ren, Hang Yuan, Zhongbao Chang.

**Methodology:** Qianchun Zhang, Ju Zhang, Fei Pan, Xuefang Ren, Hang Yuan, Zhongbao Chang.

**Writing – original draft:** Fenfang Wang, Qianchun Zhang, Penghui Yang, Fei Pan, Hang Yuan.

**Writing – review & editing:** Fenfang Wang, Qianchun Zhang, Ju Zhang, Penghui Yang, Fei Pan, Xuefang Ren, Hang Yuan, Zhongbao Chang.
